# Associations between non-suicidal self-injury and negative romantic relationship life events in male justice-involved adolescents

**DOI:** 10.1186/s12888-021-03408-7

**Published:** 2021-08-13

**Authors:** Boglárka Drubina, Gyöngyi Kökönyei, Melinda Reinhardt

**Affiliations:** 1 Doctoral School of Psychology, ELTE Eötvös Loránd University, Budapest, Hungary; 2 Department of Clinical Psychology and Addiction, Institute of Psychology, Faculty of Education and Psychology, ELTE Eötvös Loránd University, 46 Izabella Street, Budapest, H-1064 Hungary; 3 Child Protection Centre of Budapest and Local Child Protection Professional Services, Expert Committee, Budapest, Hungary; 4 SE-NAP2 Genetic Brain Imaging Migraine Research Group, Hungarian Academy of Sciences, Semmelweis University, Budapest, Hungary; 5 Department of Pharmacodynamics, Faculty of Pharmacy, Semmelweis University, Budapest, Hungary; 6 XIV District Medical Center, Child and Adolescent Psychiatry, Budapest, Hungary

**Keywords:** Non-suicidal self-injury, Justice-involved male adolescents, Negative life events, Impulsivity

## Abstract

**Background:**

In the current study we investigated impulsivity and negative life events in relation to non-suicidal self-injury (NSSI) in correctional settings.

**Methods:**

A total of 141 male justice-involved juveniles participated in our cross-sectional study, aged between 14 and 21 years old (M = 17.75; SD = 1.38). Data collection took place in correctional institutions in Hungary. A binary logistic regression was conducted to investigate possible associations between NSSI, impulsivity and negative life events.

**Results:**

Lifetime prevalence of NSSI was 53.9% (*N* = 76). In a binary logistic regression model, only negative romantic relationship events were significantly associated with the risk of current NSSI (OR = 1.29; 95% CI = [1.06–1.56]). Other types of negative life events (family-related, friendship-related), impulsivity, age and conviction status did not have a significant role in the model.

**Conclusions:**

The results suggest that juvenile offenders should receive additional support to manage stress that is associated with negative life events, especially problems in romantic relationships. It is essential to help young inmates to find an adaptive way of reducing stress caused by negative relationship life events.

**Supplementary Information:**

The online version contains supplementary material available at 10.1186/s12888-021-03408-7.

## Background

Non-suicidal self-injury (NSSI) is defined as the intentional, direct destruction (scratching, bruising, cutting, burning, biting) of one’s own body tissue without suicidal intent [1]. Over the past few years, the volume of research literature on NSSI has increased, since it is a widespread behavioural problem especially among adolescents [2, 3]. The adolescent focus of this research field is no coincidence: NSSI typically emerges between the ages of 12 and 14 [4]. The prevalence of NSSI is high in non-clinical adolescent populations (between 17 and 38%) [5–7] and even higher in clinical adolescent samples (40 to 80%) [4].

Although, there are investigations in prisons related to different types of self-injury (e.g., self-harm with suicidal intent), research focusing on NSSI remains limited. Most of the studies measure self-injurious behaviours that include suicidal ideation or attempted suicide as well [8–10], making it difficult to compare different papers in the same field of research. Furthermore, most of the studies include adult prisoners [10], therefore our knowledge of NSSI among juvenile prisoners is limited.

To clarify the term NSSI, we compare it to two other terms that are frequently used in the literature. The first one is SIB (self-injurious behaviours), which is used in two different contexts. On one hand, it is used as an umbrella term to describe any kind of self-injurious behaviour (e.g., cutting, swallowing sharp objects, having suicidal thoughts) [11, 12]. On the other hand, as coined by Favazza [13], SIB is used exclusively for self-injurious acts that are not motivated by suicide. Thus, according to the definition of NSSI used in the present study, SIB includes more behaviours than NSSI. The second term to be clarified is DSH (deliberate self-harm) which was suggested by Pattison and Kahan [14]. When the term was created, authors included fatal cases in the investigation as well. In our research, we focus only on non-suicidal self-injury (NSSI): deliberate self-injurious acts without suicidal intent.

### NSSI in correctional settings

NSSI is a serious health concern for adult offender populations [15]. Compared to the general population, the prevalence of NSSI is higher in prisons, suggesting that the prison environment may foster this behaviour [15].

A study [9] which included only one type of NSSI (cutting), demonstrated that the prevalence of NSSI (cutting) among young male offenders (14.7%) was over four times higher than the prevalence of NSSI (cutting) among undergraduate males (3.5%) [9]. Another study, which measured lifetime prevalence of self-harm (without suicidal intent), found that 28.9% of male inmates aged between 15 and 20 had already harmed themselves before [16]. The most frequent methods of self-harm (without suicidal intent) are cutting, head banging, kicking or punching things [16].

A recent meta-analysis found that being younger than 30 years old (OR = 2.0; CI = [1.4–2.9]) is a significant risk factor for self-injury (including suicide attempt as well) [10].

The new conditions and deprivation which recently imprisoned adults must face can increase the level of psychological distress, especially the risk of physical and mental health problems [17]. Therefore, one possible risk factor for engaging in DSH (with low suicidal intent) in correctional settings may be the initial period of imprisonment and the uncertainty prisoners go through during the remand (also known as pretrial detention or provisional detention) [18]. Another study found the opposite. Being convicted and sentenced was a significant predictor of NSSI between the age of 16 ang 64 (OR = 1.59; CI = [1.15, 2.21]), while being on remand did not predict such behavior [19]. To clarify the link between the conviction status and NSSI we include conviction status in the analyzed model as an independent variable.

### NSSI and impulsivity

According to our knowledge, there is no impulsivity specific NSSI theory in the literature, however impulsivity and NSSI frequently co-occur, and impulsivity has been suggested being theoretically important in the connection to NSSI [20–22].

In the 1980s, Pattison and Kahan [14] suggested that self-injury could be explained by dysfunctional impulse control. They proposed a new diagnostic category, which they called Deliberate Self-Harm Syndrome (DSH), in which people are unable to resist the urge to harm themselves. Based on DSH, the dynamics of the co-occurrence of NSSI and impulsivity is the following: since NSSI can be an effective emotion regulation strategy in the short term in case of negative emotions [23], the likelihood of NSSI is higher among people with higher levels of impulsivity. In their case, the immediate emotion-regulating effect of NSSI overwrites its possible negative effects (e.g., having bruises, stigmatization) in the long term [23, 24].

Another aspect of the co-occurrence of NSSI and impulsivity is the impulsive nature of NSSI. Nock and Prinstein [25] found that most people who engage in NSSI think for less than 5 min about harming themselves before doing so, which also supports the link between impulsivity and NSSI. Nock [26] also states that people who are more impulsive may be at greater risk of NSSI, since most of these acts do not require any planning or preparation, they are more likely to choose this kind of dysfunctional coping strategy than any other.

According to more recent research, there is a significant association between NSSI and impulsivity. Moreover, impulsivity is often considered as a risk factor for NSSI [27, 28]. A recent meta-analysis found that impulsivity significantly rises the odds of self-injury (studies included in the analysis examined suicide attempts and self-injury with suicide intent as well) among adult prisoners (OR = 4.0; CI = [2.6–6.3]; *p* < 0001) [10].

A remarkably large part of scientific literature agrees on the significant relationship between impulsivity and NSSI, however, some studies raise attention on the mixed results of longitudinal studies that investigate the associations between NSSI and impulsivity, and studies that control other variables (e.g., gender, child maltreatment, disordered eating, depression, alcohol use) in the link between impulsivity and NSSI [28–32]. The association between NSSI and impulsivity is not always significant or particularly strong [27]. Due to inconsistency in current literature, it is important to clarify the link between impulsivity and NSSI in the population of juvenile delinquents, because the level of impulsivity is clearly higher in samples of young offenders than in normative adolescent samples [22, 33].

### NSSI and negative life events

Most of the studies that have investigated stressors in the prediction of NSSI involved seriously adverse life events (e.g., sexual abuse, maltreatment, emotional abuse, drug abuse in the family) between NSSI and no-NSSI groups, with the NSSI group reporting more events [34].

Some evidence shows that everyday stressors (negative life events) play an important role in NSSI as well [26]. They can be conceptualized as proximal risk factors that explain NSSI [35, 36]. More negative life events were experienced by young people engaging in NSSI or SIB (self-injury that also includes suicidal intent) than their non-injurer peers [36–39]. Furthermore, they have an accumulative effect: more negative life events can cause more stress [40]. These everyday stressors are linked with both minor (e.g., self-hitting) and severe (e.g., cutting and burning) NSSI episodes [40]. Two longitudinal studies found that negative life events predict NSSI and SIB (self-harm that also includes suicidal intent) longitudinally in adolescence [36, 41].

During adolescence interpersonal relationship life events seemed to have a significant role in mental health [42]. Peer and family relationship difficulties are associated with SIB and DSH behaviours as well [36, 40, 43]. In a sample of Hungarian high-school students, D-SIB (direct self-injurious behaviour without suicidal intent) was associated with family problems, trouble with police or law and difficulties with romantic/sexual relationships [44].

We have very little information about the associations between negative life events and NSSI in correctional settings. One study that investigated suicidal and self-harming behaviour (SSH including self-injury with suicidal intent) found that young offenders with SSH reported more severe childhood traumas, psychological distress, and interpersonal problems (e.g., lack of friends) than non-SSH young offenders. Past physical abuse and current psychological distress were also significant risk factors for self-injury ideation [16]. In a high-security hospital, the most common (42%) predictor of NSSI was interpersonal conflict among adult offenders [45]. Another study that also involved adult prisoners found that 59% of inmates reported at least one childhood family history risk factor and were 4.05 times more likely to have an incident of NSSI [46]. Similarly, maltreatment in childhood and family dysfunction discriminated the group of DSH (self-injury without suicidal intent) inmates from the rest of the inmates [47].

People who engage in NSSI suffer from a lower ability of tolerating distress and from a dysfunctional emotion regulation when facing stressful life events [48, 49]. In this case, negative life events can be viewed as proximal risk factors for NSSI [39]. NSSI can be experienced as an effective emotional regulation strategy in the short term under the influence of negative emotions (that can be caused by negative life events) in case of high level of impulsivity [23]. In conclusion, people who are at higher risk of NSSI may share some intrapersonal (e.g., impulsivity) and interpersonal (e.g., poor problem-solving skills) vulnerabilities [39, 50]. Both these vulnerabilities and negative life events (especially at interpersonal domains) may trigger a maladaptive emotional regulation strategy, like NSSI.

Exploring negative life events that typically occur in adolescence in a probably highly impulsive population of juvenile delinquents can provide us precious information about the characteristics and correlates of NSSI which can facilitate the planning of future prevention and intervention strategies. Furthermore, Liu and his colleagues [39] suggest that documenting stressful life events gives us information not only about who, but also about when people are at imminent risk of NSSI.

### Current study

The overall aim of our research was to collect information about NSSI (e.g., frequency and types of NSSI) in the population of juvenile delinquents with a focus of the associations between NSSI, impulsivity and negative life events.

We hypothesized that, in a binary logistic regression, both impulsivity and negative life events would be significantly associated with NSSI. Some researchers have also identified a link between negative life events and impulsivity. Hayaki and her colleagues [51] identified a significant positive link between impulsivity and negative life events among drug abusers.

According to earlier research, people who engage in NSSI tend to be more impulsive [23, 28], therefore impulsivity is a relevant independent variable in the binary regression model. Based on previous research we expect that the number of negative life events is associated positively with NSSI. There are fewer papers focusing on everyday stressors and negative life events, although some studies have found significant associations between self-injury (NSSI, DSH, SIB) and adverse life events both cross-sectionally and longitudinally [36, 38, 40]. In the current study, we added three types of independent variables to the binary regression model regarding negative life events: those connected to the family and parents, those connected to romantic relationships, and those connected to friendships.

Age and conviction status (already convicted or being in pretrial detention) were also used as independent variables. Younger age is associated with self-injury (both with and without suicide intent) [10, 52]. Previous findings show inconsistent results regarding whether the conviction status is associated with self-injury or not [15, 19].

## Method

### Participants and procedure

Due to the characteristics of the population, male juvenile delinquents are overrepresented in detention centres compared to female juvenile delinquents [53], therefore our sample comprises young male offenders living in three different juvenile detention centres in the 2017/18 academic year in Hungary. A total of 152 adolescents participated voluntarily, although 11 (7.2%) participants had to be excluded because of incomplete responses. We accepted a maximum of five missing responses per scale. The dropout rate due to too many missing values was 5% (*n* = 7) in the case of the Barratt Impulsiveness Scale [54], and 3% (*n* = 4) in the case of the Adolescent Life Event Questionnaire [55]. In case of less than 5 missing responses per scale linear interpolation method was used to impute the missing data [56]. According to the linear interpolation method, the missing value is computed based on the last complete observation value before the missing data and the first complete observation value after the missing data [57].

In the final sample there were 141 respondents. The youngest participant was 14 years old (*n* = 1), and the oldest participants were 21 years old (*n* = 2) (M_age_ = 17.75, SD = 1.38). Eighty of them (56.7%) already had a conviction, while the rest (43.3%; *n* = 61) were in pretrial detention. The majority (58.9%; *n* = 83) were attending primary school, 38 participants (26.9%) were attending high school or vocational school, and 17 participants (12.1%) were not attending school at the time of the study.

The average length of time spent in the current correctional institution was 12 months (SD = 10.84). Most of the adolescents (82.3%; *n* = 116) were in regular touch with their families.

All aspects of the study were ethically approved by the Institutional Review Board of ELTE Eötvös Loránd University, Faculty of Education and Psychology. All the correctional institutions provided written consent to participate in the research. The recruitment started afterwards. Inclusion criteria was the typical age of the incarceration’s onset, 14 years old. Initially, oral information was provided about the procedure for the potential participants, after which the respondents and their guardians or representatives were asked to give their written informed consent. Adolescent were informed that participation is anonymous and voluntary. None of the eligible justice-involved juveniles refused to participate. Respondents who expressed a willingness to complete the questionnaires attended the data collection sessions in small groups of two to seven people. Participants completed the questionnaires in their mother tongue, on tablets, under the supervision of trained investigators. No staff from the detention centres were present. Participation in the study was voluntary and anonymous.

After completing the questionnaires, participants were invited to ask questions and share their thoughts about the research topic. Our trained investigators replied to the questions and emphasized that participants could turn to the institutions’ psychologists if they were dealing with difficulties like those featured in the questionnaires.

### Measures

#### Self-injury questionnaire – treatment related (SIQ-TR)

Claes and Vandereycken [58] developed the SIQ-TR to measure deliberate self-injurious behaviour (SIB) without suicidal intent. The questionnaire focuses on nine features related to five types of self-harm (scratching, bruising, cutting, burning, and biting, with an opportunity to include one type not already mentioned). The nine features are: injured body part; frequency of self-injury in the previous month; daily frequency; frequency of feeling pain during self-injuring (1 = never, 4 = always), and intensity of pain (1 = none, 5 = very strong); along with four attitudinal aspects of self-injury on a five-point scale (1 = never, 5 = always): advance planning, consciousness, taking care of wounds, and concealing the act. Feelings before and after the self-injurious act are also investigated (e.g., glad, anxious, sad). The final feature is the function fulfilled by self-injury (e.g., to feel some pleasure). By using factor analysis, the authors identified three factors as different functions of NSSI. Social Positive Reinforcement (α = .68), Automatic Positive/Negative Reinforcement (α = 0,65; α = .70) [58]. In the current study reliability of functions are acceptable (Social Positive Reinforcement α = .69; Automatic Positive Reinforcement α = .79; Automatic Negative Reinforcement α = .77). In the Hungarian version of the SIQ-TR, there are six possible answers to the question “How long ago did you scratch/bruise/cut/burn/bite yourself?” These are: less than a week ago (added by us), 1 week ago, 1 month ago, several months ago, over a year ago, never. We considered current self-harmers those who chose one of the first four answers. The reason for this grouping was that we were investigating the association between NSSI and negative life events that had happened in the last 6 months. There can be an association between the two only if they occur at approximately the same time. Participants who engaged in self-harm over a year ago are labelled as life-time self-injurers. In this study we compare those who currently engage in NSSI to the rest of the participants (lifetime NSSI and participants who have never engaged in NSSI).

#### Barratt impulsiveness scale (BIS-11)

This scale measures three aspects of impulsivity: attentional (cognitive) impulsiveness, motor impulsiveness, and non-planning impulsiveness. Patton, Stanford, and Barratt [54] validated their 30-item questionnaire among community, clinical, and offender samples. All items are answered on a four-point Likert scale (1 = rarely/never, 2 = occasionally, 3 = often, 4 = almost always/always). The higher the total score, the higher the level of impulsivity. In the current study, the internal consistency was .74, which is consistent with the results (α = .80 in a sample of adult offenders) obtained by Patton and his colleagues [54]. Consistency of the subscales in the present study were motor impulsiveness α = .61, attentional impulsiveness α = .44, and non-planning impulsiveness α = .53. As these scores are very low, impulsiveness subscales were not included in the statistical analysis. In a study that investigated the psychological properties of the BIS-11 in a sample of Portuguese juvenile delinquents demonstrated that the 3-factor second-order factorial structure did not present sufficiently good fits [59].

#### Adolescent life events questionnaire (ALEQ)

Hankin and Abramson [55] used this questionnaire to measure typical stressors and negative life events occurring in adolescence. The original questionnaire comprises 70 items. We adjusted the questions to the specific sample, using the following three subscales: family and parents (e.g., “Your parents grounded you.”), romantic relationship events (e.g., “Boyfriend/girlfriend broke up with you, but you still want to go out with them.”), and friendship events (e.g., “Had an argument with a close friend.”). We excluded the school subscale as some delinquents do not go to school in detention, furthermore classes provided by the detention centre are different from normal school environment. We used 39 items to investigate whether these negative life events had been experienced in the previous 6 months. The internal reliability of the ALEQ was .84, which is consistent with the results obtained by Hankin and Abramson (α = .94) [55]. Test–retest reliability over 2 weeks was .65 [55]. In our study reliability of the subscales are the following: family and parents related events subscale α = .69; romantic relationship events subscale α = .69; and friendship events subscale α = .69. Reliability of the subscales are not reported in the original study.

### Data analysis

The SPSS 26 program was used for the analysis. The significance level in the study was taken as .05. In the current study, the dependent variable is NSSI.

The first independent variable is impulsivity. Most of the studies that use the Barratt Impulsiveness Scale indicate summed values in the analyses [14, 59]. On several occasions, respondents in our sample failed to give an answer for a couple of the items (see dropout rates in *Participants and Procedure*), therefore data imputation was used to be able to produce results that are comparable with previous research (see data imputation details in *Participants and Procedure*). Due to low reliability of impulsivity subscales, we will investigate impulsivity as a structure in association with NSSI.

The second independent variable are negative life events that typically occur in an adolescent’s life. We used both the summed number of negative life events that had happened during the last 6 months (maximum 39) and the summed number of each type of negative life events (family and parent events: maximum 11; romantic relationship events: maximum 11; friendship events: maximum 17). We had four independent variables connected to negative life events.

Age and conviction status (1 = already convicted; 2 = pretrial detention) were also used as independent variables.

First, we analysed the prevalence and types of NSSI behaviour. Independent samples t-tests were conducted afterwards to explore possible differences between the groups of self-injurers and non-injurers in terms of impulsivity (BIS-11), negative life events (ALEQ and its subscales), time spent in custody, and whether the respondent had already been convicted. Cohen’s d effect size was also reported.

Finally, a binary logistic regression analysis was carried out to test our model. We used a block wise entry method in the binary regression and analysed three blocks. The significance of each block of independent variables was tested to show whether the addition of a new block contributed significantly to the model. In the first step, Block 1 contained the three ALEQ subscales (family, friends, romantic relationships). In the second step, Block 2 contained the summed BIS-11 score. In the third step, Block 3 contained age and conviction status.

## Results

### Descriptive statistics

#### Descriptive statistics of NSSI

In the present sample, 31.9% of the participants (*n* = 45) reported at least one current episode of NSSI. Lifetime prevalence was even higher 53.9% of the participants (*n* = 76) had already engaged in NSSI during their life. The most common method of NSSI in our sample was scratching (53.3%; *n* = 24), followed by bruising (48.8%; *n* = 22), cutting (37.7%; *n* = 17), burning (2.8%; *n* = 4), and biting (2.1%; *n* = 3). Most participants used either one method (57.8%; *n* = 26) or two methods (24.5%; *n* = 11).

#### Descriptive statistics of the scales

In the previous 6 months, on average 13 (M = 12.63; SD = 6.45) of 39 negative life events had been experienced by participants. The average impulsivity score was 64.33 points (SD = 10.1) (Table 1).

**Table 1:** Descriptive statistics for variables, scales, and Pearson correlation matrix

	Min.	Max.	M	SD	1.	2.	3.	4.	5.	6.	7.	8.
1. BIS-11	40	92	64.33	10.10	–							
2. ALEQ	0	37	12.63	6.45	.15	–						
3. ALEQ-family	0	11	3.80	2.51	.01	.69**	–					
4. ALEQ-relationships	0	11	3.68	2.46	.17*	.81**	.37**	–				
5. ALEQ-friendships	0	17	5.14	3.20	.16	.84**	.32**	.58**	–			
6. age	14	21	17.70	1.40	.07	.08	.08	.06	.10	–		
7. conviction status^1^	1	2	1.43	0.49	.01	.00	.00	.15	.00	.31**	–	
8. NSSI^2^	1	2	1.32	0.46	.09	.24**	.06	.35**	.15	.02	.01	–

*Note*. **p < 0.01; *BIS-11* Barratt Impulsiveness Scale; *ALEQ* Adolescent Life Events Questionnaire; ^1^ coded as: 1 = already convicted, 2 = pretrial detention; ^2^ coded as: 1 = no NSSI, 2 = current NSSI

### Binary logistic regression analysis

Table 2 presents the results of the binary logistic regression analysis that was used to determine whether negative life events, impulsivity, age, and conviction status could explain NSSI. The regression analysis comprised three steps. The results showed that only romantic relationship negative life events (OR = 1.285; *p* < .01) made a significant contribution in the explanation of the dependent variable (NSSI). Age, conviction status, impulsivity, family-related negative life events, and friendship-related negative life events did not contribute significantly to the model. A multinominal regression analysis with three types of NSSI status (never had engaged in NSSI, lifetime NSSI, current NSSI) did not show different results (see in supplementary material).

**Table 2:** Binary logistic regression analysis: explaining NSSI

	OR	95% CI	χ^2^	Nagelkerke R^2^
Block 1			15.63***	0.150
ALEQ-family	0.961	[0.816–1.132]		
ALEQ-relationships	1.285**	[1.061–1.557]		
ALEQ-friendships	1.084	[0.940–1.251]		
Block 2			15.66**	0.150
ALEQ-family	0.962	[0.816–1.134]		
ALEQ-relationships	1.283**	[1.058–1.555]		
ALEQ-friendships	1.250	[0.938–1.250]		
BIS-11	1.003	[0.965–1.043]		
Block 3			16.158**	0.155
ALEQ-family	0.964	[0.817–1.139]		
ALEQ-relationships	1.299**	[1.065–1.584]		
ALEQ-friendships	1.078	[0.932–1.246]		
BIS-11	1.005	[0.966–1.045]		
age	1.014	[0.758–1.357]		
conviction status	1.338	[0.591–3.030]		

Note. **p < 0.01; ***p < 0.001; *BIS-11* Barratt Impulsiveness Scale; *ALEQ* Adolescent Life Events Questionnaire

### Frequency of negative romantic life events

Based on the results that suggest that negative romantic life events significantly raise the odds of an NSSI episode we found it important to report the frequency of each negative romantic life event in the sample. It is showed in Table 3.

**Table 3:** Frequency of romantic relationship negative life events in the whole sample

Romantic relationship negative life events	N (%)
A boyfriend/girlfriend breaks up with you, but you still want to go out with them.	54 (38.3)
Made someone pregnant when you didn’t want to.	47 (33.3)
Had a baby that you didn’t plan or want.	20 (14.2)
Don’t have a boyfriend/ girlfriend when you want one.	50 (35.5)
Got in a fight/ argument with a boyfriend/ girlfriend	82 (58.2)
Can’t seem to please girlfriend/ boyfriend when you want to.	52 (36.9)
Girlfriend/ boyfriend criticizes you.	18 (12.8)
Can’t seem to get close to your boyfriend/girlfriend when you want to.	43 (30.5)
Found out that boyfriend/ girlfriend has been criticizing you behind your back.	30 (21.3)
Found out that boyfriend/ girlfriend has been cheating on you.	62 (44)
Did something to please you boyfriend/ girlfriend that you didn’t want to do.	61 (43.3)

Note*. N* = 141

This table contains all items of the *ALEQ* (Adolescent Life Events Questionnaire) Romantic relationship negative life events subscale.

## Discussion

According to the present research, the prevalence of NSSI was remarkably high in the sample of juvenile delinquents. In our study, the lifetime prevalence of NSSI was 53.9%. The prevalence of NSSI acts that had taken place currently was 31.9%. In the CASE study, only 3.4% of Hungarian male adolescents aged between 15 and 16 years old had attempted any kind of DSH (deliberate self-harm also including acts with the intent to die) in their life [60]. In the SEYLE study, the prevalence of NSSI was 17.1% in a Hungarian community adolescent sample [61]. However, it should also be considered that the population of juvenile delinquents is extremely vulnerable [62, 63], which can result in a higher prevalence of mental and behavioural problems.

Matsumoto and his colleagues [9] measured deliberate self-cutting (undetermined whether intent to die was a criterion or not) among young offenders and found a lifetime prevalence of 14.7%. In another study, 11.2% of the juvenile delinquents reported lifetime self-injury (without suicidal intent) [17]. Moore, Gaskin and Indig [64] found that 13.9% of young male offenders had already had at least one episode of self-injury (without the intent to die) during their life. Compared to these results, the lifetime prevalence of NSSI was higher in the current present sample, although we should consider that, for instance, Matsumoto and his colleagues [9] included only one type of self-injury. According to the systematic review of Dixon-Gordon and her colleagues [15], the prevalence of NSSI in prison settings ranges between 4.5 and 24% among young, incarcerated people. Our findings show higher prevalence. This can be due to the raise in the prevalence of NSSI in normative adolescent population during the last years [65, 66].

The most common method of NSSI in our sample was scratching (53.3%), followed by bruising (48.8%), cutting (37.7%), burning (2.8%), and biting (2.1%). Many studies have shown that male adolescents tend to use more severe methods such as hitting and burning [67]. In our research cutting is only the third most typical method of NSSI. We should bear in mind that, in correctional setting, due to strict security rules, it is very difficult to find objects that can be used for burning and cutting. In the case of scratching and bruising, no object is needed.

The status of the conviction is not a significant correlate of NSSI. Mohino and his colleagues [47] also found that the current penal situation did not discriminate between inmates with DSH (self-harm without suicidal intent) and inmates without DSH episodes among young adult prisoners.

Many studies support the hypothesis that there is a link between impulsivity and self-injury [23, 28]. This hypothesis was not confirmed in the present study. The summed score for impulsivity does not differentiate between the group of self-injurers and non-injurers. This result might seem surprising; however, we should take into consideration two aspects. First, the population of young offenders are a highly at-risk population regarding risk behaviours, or any additional psychiatric disorders [33, 68, 69], therefore, NSSI might be linked to different psychological problems, not only to high level of impulsivity. More complex patterns of correlates and risk factors should be investigated in this population. Second, it may be the case that the general level of impulsivity is higher among young offenders than among community adolescents [22, 33]. If this is so, in the present study impulsivity is not the most relevant construct for investigating psychological differences between self-injurers and non-injurers.

A generally high level of impulsivity among incarcerated youth might be linked to the uncertainty and stress experienced during incarceration. Intolerance of uncertainty is a characteristic which means to interpret ambiguous situations as stressful and threatening [70]. Intolerance of uncertainty has been linked to anxiety and distress that may result in maladaptive behaviors such as impulsive behavior [71]. For instance, uncertainty can be extremely high in the case of those who do not yet have a conviction.

There is a significant positive correlation between NSSI and the number of all negative life events that occurred in the previous 6 months, which is consistent with the literature [26, 72]. Romantic relationship negative life events seemed to have a significant association with NSSI behaviour.

In correctional settings, young offenders live under very strict rules. The number and frequency of visits are limited, thus even if the offenders have girlfriends, they can meet only very rarely. A recent meta-analysis found that the lack of visits can be a significant risk factor (OR = 2.3; CI = [1.5–3.5]) for self-injury (including suicide attempt as well) among adult offenders [10]. Young offenders live in an enclosed world, separated from the other sex. This can make normative sexual development extremely difficult. During adolescence, feelings of sexual arousal increase, and meeting members of the other sex is very important at this age [73]. In prison conditions, adolescents are unable to satisfy either their romantic emotional needs or their sexual needs. This is highly likely to be a source of frustration and emotional distress (see more information about the importance of peer romantic relationships during adolescence in [74]). It may be the case that juvenile delinquents are unable to handle this stress, which prompts them to find a non-adaptive coping mechanism such as NSSI, for instance. This may explain why relationship-related negative life events are significantly correlated with NSSI.

Most of the studies that have investigated the link between NSSI, and life events have referred to significant traumas [75], but not to everyday stressors or negative life events. Our research demonstrates that relationship-related negative life events play a significant role in the lives of juvenile delinquents.

We are not aware of any other research that has investigated NSSI and its relationship with negative life events and impulsivity in correctional settings.

### Clinical implications

In general, the level of psychopathology and psychological difficulties are high in justice involved adolescents [33, 63, 68], therefore the treatment of NSSI requires a complex methodology that focuses on comorbid psychological disease as well. Different risk factors (e.g., hopelessness, depressive symptoms, exposure to peer NSSI) [27] should be considered to provide the most appropriate psychological care for young delinquents. Structured psychotherapeutic approaches that focus on collaborative therapeutic relationships and motivation for change while directly keeping in focus the NSSI behavior itself seem to be the most effective in reducing NSSI [76].

Cooperation and an ongoing communication are needed between mental health stuff and security stuff to monitor and to detect early signs of NSSI. Policies that outline responsibilities for staff such as care planning and monitoring is necessary [77].

This study highlights the important role of professionals working in juvenile detention centers. It might be important for them to focus on and ask about negative life events, especially those connected to romantic relationships as indicators of risk for NSSI.

In general, learning adaptive and functional coping strategies to deal with (interpersonal) stress seems to be an important means of prevention and treatment of NSSI in correctional settings. Furthermore, improving interpersonal relationships (mostly family and romantic relationships) might be essential as well when planning the strict daily routine and rules of family visits (e.g., longer, and more often visits with partners and family members).

### Limitations and suggestions for future research

Despite the value of the current study, it does have limitations. The first limitation is the weak reliability of the subscales in the Barratt Impulsiveness Scale. Some of the BIS-11 items were inappropriate for a correctional setting (e.g., “I plan trips well ahead of time”; “I save regularly”). Although the researchers asked participants to respond to the questions as they would in general (rather than in a correctional setting), it is possible that imprisonment resulted in inconsistency in the responses. There may also have been problems with the factor structure of the BIS-11. Pechorro, Maroco, Ray and Goncalves [59] investigated the psychological properties of the BIS-11 in a sample of juvenile delinquents. Using confirmatory factor analysis, they found that the three-factor second-order structure (attentional/cognitive impulsiveness; motor impulsiveness; and non-planning impulsiveness) did not offer sufficiently good fits. In general, total scores showed acceptable internal consistency (α = .84). One important question that remains to be investigated in the future is whether Barratt’s three-factor model of impulsivity is the optimum choice for application in correctional settings. It may be the case that, in correctional settings, other aspects of impulsivity are more relevant (e.g., sensation seeking as conceptualized by Whiteside & Lynam [78]). Further research is needed to identify which aspects of the multidimensional construct of impulsivity are the most relevant in correctional settings, and which tool or questionnaire is the most appropriate. Impulsivity might be linked to uncertainty [71]. Another limitation in the study is the time frame used in the grouping of the NSSI involved group. In the Hungarian version of SIQ-TR [58] there are six possible answers to the question “How long ago did you scratch/bruise/cut/burn/bite yourself?” These are: “less than a week ago”, “one week ago”, “one month ago”, “several months ago”, “over a year ago”, “never”. We considered current self-injurers those who chose one of the first four answers. The answer “several months ago” can mean more than 6 months, that is measured in the Adolescent Life Events Questionnaire [55], therefore it might be possible that an NSSI episode marked with “several months ago” occurred prior to the measured life events which can lead to distortion in the results.

NSSI is an intimate topic that is difficult to talk or write about. In our experience, there were popular and accepted forms of NSSI in correctional settings, as well as less popular forms. Those who bruise themselves, for example (e.g., by punching the wall), are regarded as strong according to correctional norms. Participants may have been more unwilling to admit less popular forms of NSSI (e.g., biting).

Since the current study is cross-sectional, we were unable to infer any causal associations. In the future, longitudinal studies are required to better understand the risk factors for NSSI in male justice-involved adolescents.

A possible direction for future research is to discover the protective factors that can help young male offenders to find an adaptive way for releasing their stress. In our research we asked participants at the end of the survey to write a significant positive memory or experience that happened in their life. Out of 109 participants who answered this question, most of them (41.2%; *n* = 45) wrote a memory which is connected to their family (e.g., “We celebrated my birthday with my family.”; “Staying at home with my parents.”; “The birth of my little brother.”; “Talking to my siblings.”). Tatnell and her colleagues [79] found that perceived family support is an important safeguard against NSSI. Family related negative life events are not associated with NSSI in our study, but it is possible and should be consider in future research, that positive family events might help in coping with stress that come from romantic relationships.

## Conclusion

The aim of the present study was to investigate NSSI among male juvenile delinquents and to establish a model with possible correlates of NSSI. According to our findings, lifetime prevalence of NSSI was remarkably high. A significantly higher number of negative life events occurred among self-injurers compared to non-injurers. The average level of impulsivity was generally high in the sample, although there was no significant differentiation between self-injurers and non-injurers. Furthermore, the results of the study suggest that romantic relationship negative life events (e.g., breaking up with a girlfriend) are associated with the risk for NSSI among male juvenile delinquents. Despite of previous results, impulsivity is not associated significantly with the risk for NSSI.

## Supplementary Information

**Additional file 1: Table A1.** *Multinominal regression analysis: explaining NSSI* ^*a*^ *. (DOCX 18 kb)*

## Data Availability

The datasets used and/or analysed during the current study are available from the corresponding authors on reasonable request.

## Abbreviations

ALEQ: Adolescent life events questionnaire
BIS-11: Barratt Impulsiveness Scale
DSH: Deliberate self-harm
D-SIB: Direct self-injurious behaviour
NSSI: Non-suicidal self-injury
SIB: Self-injurious behaviours
SIQ-TR: Self-injury questionnaire – treatment related
