# Supplementary material for: Associations between non-suicidal self-injury and negative romantic relationship life events in male justice-involved adolescents
Source: BMC Psychiatry. 2021 Aug 13;21:401. doi: 10.1186/s12888-021-03408-7 (PMC8361803; doi:10.1186/s12888-021-03408-7)
Supplement: Supplementary file 1 — Additional file 1: Table A1. Multinominal regression analysis: explaining NSSI a . (DOCX 18 kb) [file 12888_2021_3408_MOESM1_ESM.docx]

| **Table A1**  *Multinominal regression analysis: explaining NSSI^a^* | | | | | | | | | | | | | | | | | | | | | | | | | | |  |  |  |
| --- | --- | --- | --- | --- | --- | --- | --- | --- | --- | --- | --- | --- | --- | --- | --- | --- | --- | --- | --- | --- | --- | --- | --- | --- | --- | --- | --- | --- | --- |
|  | | Estimate | | | | SE | | | | OR | | 95% CI | | | | | χ^2^ | | | | | Nagelkerke R^2^ | | | | |  |  |  |
| *lifetime NSSI^b^* | | |  | | | | |  | | |  | |  | | | | | 28.14** | | | | | 0.20 | | | | |  |  |
| ALEQ-family | | | -0.07 | | | | | 0.28 | | | 0.92 | | | | [0.533 – 1.160] | | | | |  | | | | |  | | |  |  |
| ALEQ-relationships | | | 0.12 | | | | | 0.17 | | | 1.13 | | | | [0.616 – 2.104] | | | | |  | | | | |  | | |  |  |
| ALEQ-friendships | | | -0.09 | | | | | 0.27 | | | 0.91 | | | | [0.534 – 1.558] | | | | |  | | | | |  | | |  |  |
| ALEQ | | | 0.09 | | | | | 0.27 | | | 1.10 | | | | [0.644 – 1.885] | | | | |  | | | | |  | | |  |  |
| BIS-11 | | | -1.02 | | | | | 0.63 | | | 0.36 | | | | [0.104 – 1.245] | | | | |  | | | | |  | | |  |  |
| age | | | 0.15 | | | | | 0.14 | | | 1.17 | | | | [0.874 – 1.568] | | | | |  | | | | |  | | |  |  |
| conviction status | | | -0.28 | | | | | 0.44 | | | 0.75 | | | | [0.313 – 1.807] | | | | |  | | | | |  | | |  |  |
| *current NSSI^c^* |  | | | |  | | | |  | | | |  | | | | |  | | | | |  | | | | |  |  |
| ALEQ-family | | | | 0.22 | | 0.22 | | | | 1.24 | | | | [0.799 – 1.953] | | | | |  | | | | |  | | | | | |
| ALEQ-relationships | | | | 0.653 | | 0.30 | | | | 1.91* | | | | [1.064 – 3.458] | | | | |  | | | | |  | | | | | |
| ALEQ-friendships | | | 0.388 | | | | 0.24 | | | | 1.47 | | | | | [0.920 – 2.361] | | | | |  | | | | |  | | |  |
| ALEQ | | | -0.29 | | | | 0.23 | | | | 0.74 | | | | | [0.464 – 1.186] | | | | |  | | | | |  | | |  |
| BIS-11 | | | 0.45 | | | | 0.69 | | | | 1.57 | | | | | [0.400 – 6.193] | | | | |  | | | | |  | | |  |
| age | | | 0.005 | | | | 0.02 | | | | 1.00 | | | | | [0.961 – 1.050] | | | | |  | | | | |  | | |  |
| conviction status | | | 0.38 | | | | 0.50 | | | | 1.46 | | | | | [0.547 – 3.910] | | | | |  | | | | |  | | |  |

^Note. *p<0.05; **p<0.01; ***p<0.001; BIS-11=Barratt Impulsiveness Scale; ALEQ=Adolescent Life Events Questionnaire; a. reference category is NSSI never = never had engaged in NSSI; b. lifetime NSSI = engaged in NSSI over a year ago, but currently do not engage in NSSI; c. current NSSI = engaged in NSSI less than a week ago, one week ago, one month ago or several months ago.^

A multinominal regression analysis was conducted to determine whether a more sensitive grouping of NSSI leads to different results.

NSSI was grouping as the following: no history of NSSI (never had engaged in NSSI), lifetime NSSI (engaged in NSSI over a year ago, but currently do not engage in NSSI) and current NSSI (engaged in NSSI less than a week ago, one week ago, one month ago or several months ago). The results show that there is only one significant independent variable in the model, negative romantic relationship life events, which differentiate significantly between those who currently engage in NSSI and those, who never had engaged in NSSI. The results are consistent with results of the binary regression model.
